# Development, Characterization, and Exploitation in Food Systems of Functional Ingredients Obtained from Artichoke By-Products Phenolic Extracts

**DOI:** 10.3390/molecules30071514

**Published:** 2025-03-28

**Authors:** Francesco Iervese, Arianna Paluzzi, Michela Cannas, Giulia D’Alessio, Antonio Piga, Carla Di Mattia

**Affiliations:** 1 Department of Bioscience and Technology for Food, Agriculture and Environment, University of Teramo, Via Balzarini 1, 64100 Teramo, Italy; fiervese@unite.it (F.I.); gdalessio@unite.it (G.D.); 2 Department of Agricultural Sciences, University of Sassari, Viale Italia, 07100 Sassari, Italy; mcannas@uniss.it (M.C.); pigaa@uniss.it (A.P.)

**Keywords:** o/w emulsions, oxidation, technological functionality, polyphenols, release, in vitro digestion

## Abstract

The study aimed to assess the technological properties of six ethanolic phenolic-rich extracts derived from artichoke bracts, stems, and leaves using different extraction methods (maceration and ultrasonic-assisted extraction—UAE) for the formulation of oil-in-water emulsions in which pea protein concentrate served as an emulsifier. To this aim, the extracts were tested for their surface properties and their effect on the colloidal and antioxidant properties in emulsions. The extracts reduced the surface tension at the water/air interface in a dose-dependent manner, with the leaf extract obtained by UAE displaying the highest surface activity. In emulsions, the extracts increased oil droplet size and induced flocculation while being able to delay oxidation, as indicated by the induction period significantly higher compared to the control. In the last part of the work, encapsulation by spray-drying was explored on a selected leaf extract, and its release behavior in an enriched vegan mayonnaise was tested by in vitro digestion. The encapsulation influenced the release of phenolic compounds during simulated gastrointestinal digestion of the enriched vegan mayonnaise, demonstrating promising protective effects in the gastric environment and promoting a predominant release during the intestinal phase, potentially enhancing the absorption and bio-accessibility of the phenolic compounds.

## 1. Introduction

The agri-food sector exhibits several shortcomings in waste management, with approximately 14% of global food production being lost between the point of harvest and retail sale [1]. This phenomenon is particularly pronounced in the fruit and vegetable supply chain, given the large volume of production and relatively short shelf life. It poses both environmental and economic challenges, considering that these waste products often contain bioactive compounds that could be valorized and further exploited [2]. For instance, by-products from various parts of fruits and vegetables contain a diverse range of bioactive compounds, including phenolic acids, flavonoids, anthocyanins, carotenoids, and vitamin C. These bioactive compounds not only have the potential to be utilized as ingredients for developing new functional food products but also serve as valuable components in nutraceuticals for medicinal and pharmaceutical applications [3].

Artichoke (*Cynara scolymus* L.) is an herbaceous perennial plant belonging to the family of Asteraceae. Its crop is widely distributed, with a cultivation area of 113,058 ha, and a production of 1.58 million tons, of which 0.61 are produced in Europe. Italy is the largest artichoke-producing country in the world, with a cultivated area of just over 38,000 ha and a total production of flower heads equal approximately to 378,110 tons [4]. Artichoke is primarily cultivated for the production of its inflorescence, in which the innermost part constitutes the edible component. Therefore, the rest of the plant represents a by-product, amounting to approximately 80–85% of the plant’s weight [5,6]. These by-products are formed mainly by leaves, stems, and external bracts rich in bioactive compounds, such as dietary fiber (inulin) and antioxidant compounds [7]. Notably, these parts contain phenolic compounds, including luteolin (a flavone), cynaroside, scolymoside (flavonoid glycosides), and additionally, phenolic acids such as cynarin, caffeic, coumaric, hydroxycinnamic, and ferulic acids [8]. Therefore, the valorization of such waste products involves several potential benefits, from the economic and environmental viewpoints as well as from the nutritional and technological ones. Moreover, the presence of phytochemical compounds associated with positive effects on human health, such as the protection of cells from oxidative stress and the control of cholesterol levels, underscores the importance of this approach [9,10]. It is thus clear that developing suitable and efficient methods for the recovery and isolation of functional and bioactive components from food by-products is crucial in this context [11], and the same can be said for their exploitation in food matrices. The adoption of green approaches for recovering phenolic compounds can facilitate the production of functional ingredients with a reduced environmental impact compared to traditional methods. Bioactive compounds, indeed, can be extracted using both conventional and non-conventional solid–liquid methods. Traditional techniques like Soxhlet extraction, maceration, and hydro-distillation rely on solvents, heat, and stirring but have drawbacks such as long processing times, high solvent costs, low selectivity, and potential thermal degradation of sensitive compounds [12]. To overcome these issues, non-conventional methods like ultrasound-assisted extraction (UAE) have been developed. UAE enhances mass transfer through cavitation, reducing extraction time, energy consumption, and solvent use. Factors such as extraction time, temperature, solvent type, and solid–solvent ratio also influence phenolic compound recovery. For example, in the study carried out by Cannas et al. (2023) [12], phenolic compounds were extracted from artichoke waste using ultrasound-assisted extraction. This method allowed to achieve extraction yields comparable to those obtained through maceration but required significantly less solvent and time. Concerning their potential exploitation, extracts from artichoke by-products found application in various matrices, including the enrichment and functionalization of bakery products with fiber concentrates [13], the inhibition of different enzyme activities in vitro [14], as well as the improvement of the oxidative stability of beef patties [15].

Furthermore, extracts from vegetable waste products can exhibit promising technological functionality in multiphasic systems like oil-in-water emulsions, which are known to be complex and thermodynamically unstable matrices that can undergo various physical and chemical instability phenomena [16]. Phenolic-rich extracts were shown to exert strong antioxidant activity in both bulk and emulsified systems, thanks to their amphiphilic nature and their capacity to adsorb on the oil/water interface where the oxidative phenomena occur [17]. Indeed, regarding oxidative stabilization, in recent years there has been a growing interest in the possibility of using interfacial antioxidant compounds to inhibit lipid oxidation in emulsions. For instance, in line with Velderrain-Rodríguez et al. (2021) [18], the inclusion of polyphenol-rich extracts derived from avocado peel and seed has been demonstrated to improve the colloidal stability of oil-in-water emulsions produced with low methoxyl-pectin (LMP) as surfactant. The exact mechanisms by which these extracts enhance stability are not yet fully understood. However, the authors suggest that phenolic compounds may act as co-surfactants. Specifically, phenolic compounds present in the aqueous phase can improve the emulsifying properties of macromolecules such as proteins and polysaccharides by interacting with the lipid phase and the emulsion interface [19]. This also resulted in a noteworthy increase in oxidative stability. Additionally, in another study, flavonoids- and limonoids-rich extract, recovered from citrus pomace, was proven to exert technological functionality by significantly affecting the dispersion degree of oil-in-water emulsions stabilized with pea isolate and by improving their chemical stability towards oxidative phenomena [17]. To date, information about the technological functionality of artichoke phenolic-rich extracts in dispersed systems is still lacking. Thus, the aim of this work was to investigate the technological properties of polyphenols-rich extracts recovered using different ethanolic solutions and extraction procedures (maceration and UAE) from bracts, stems, and leaves by-product derived from artichoke processing for the formulation of multiphasic matrices (emulsions). The experimental plan was divided into three different steps: in step 1, the surface activity and emulsifying properties of the extracts were investigated in 50% *w*/*w* oil-in-water emulsions stabilized by a pea protein concentrate; in step 2, one extract, macerated leaves (LM), was selected and encapsulated by spray-drying technology with maltodextrin as wall material; while in the final step the encapsulated extract was added to a commercial vegan mayonnaise to investigate the effect of encapsulation on the release of polyphenols during in vitro simulated human digestion.

## 2. Results and Discussion

The characterization of the artichoke extracts used in the present investigation was extensively explored in a previous study carried out by Cannas and co-authors (2023) [12], in which the content of total phenolics and flavonoids, the phenolic pattern, and the antioxidant capacity were reported, as a function of different extraction procedures (maceration vs. ultrasound-assisted extraction) and ethanol percentage in the medium (see Section 3.1 in Materials and Methods section). The authors demonstrated that irrespective of the extraction method employed, extracts derived from stems exhibited elevated levels of total polyphenols and flavonoids, followed by those derived from bracts and subsequently from leaves. Similarly, antioxidant capacity values were higher in stem and bract samples. In terms of phenolic pattern, stems and bracts extracts exhibited a comparable phenolic composition, with chlorogenic acid, 1,5-di-O-caffeoylquinic acid, and 3,5-di-O-caffeoylquinic acid being the predominant constituents. However, a high concentration of apigenin 7-O-glucoside was found in the bract extracts, whereas it was completely absent in the stem extracts. In contrast, the leaves exhibited a distinct phenolic profile, characterized by a prevalence of flavonoids, particularly luteolin-7-O-glucoside [12]. Therefore, this investigation, in which the technological functionality of artichoke phenolic-rich extracts in dispersed systems is deepened, represents a follow-up of the prior study.

### 2.1. Technological Functionality in Oil-in-Water Emulsified Systems

The phenolic-rich artichoke extracts were characterized by their technological functionality in emulsified systems. For this purpose, the surface properties and the emulsifying capacity of the extracts were studied; moreover, the enriched emulsions were characterized for their flow behavior and oxidative stability.

#### 2.1.1. Surface Properties and Emulsifying Capacity

**Figure 1:**
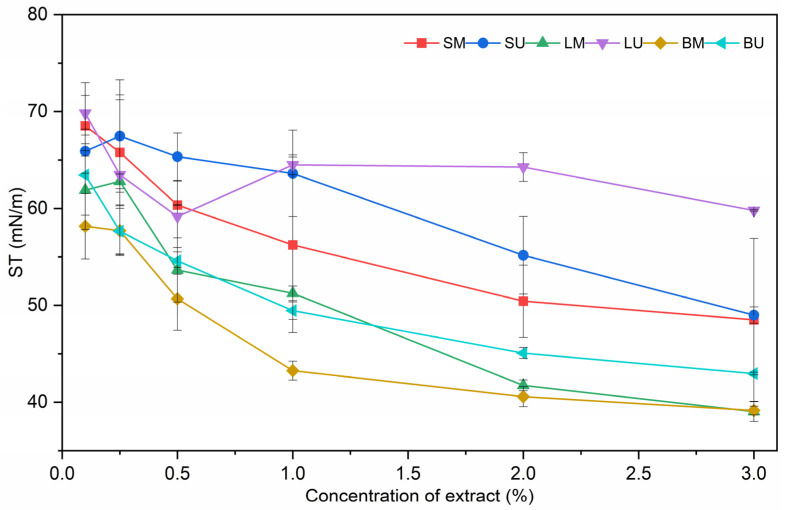
Surface tension (ST) at air/water interface of artichoke by-product extracts as a function percentage of the extracts in the aqueous phase. Acronyms: leaves macerated (LM), leaves sonicated (LU), stems macerated (SM), stems sonicated (SU), bracts macerated (BM), and bracts sonicated (BU).

Thanks to the molecular features of some phenolic compounds, which provide them with an amphiphilic behavior, phenolic-rich extracts recovered from plant by-products are often reported to lower the surface tension of aqueous solutions [17,20]. Artichoke by-products extracts were thus assessed for their surface activity at the air/water interface and the results are reported in Figure 1, in which the values of surface tension (mN/m) are shown as a function of extract concentration in the water phase (Figure 1).

**Figure 2:**
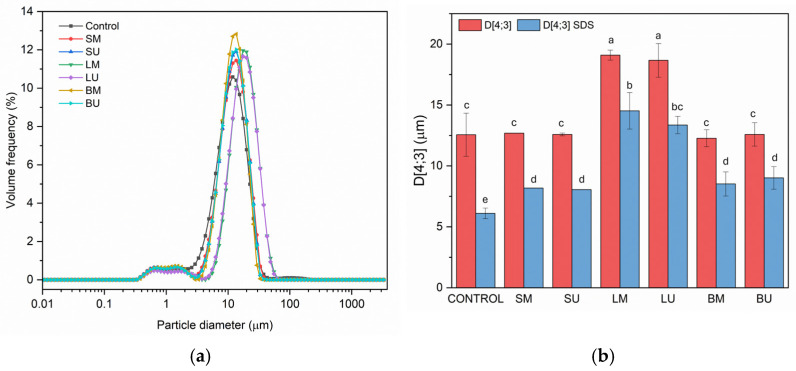
(**a**) Distribution size distribution of the o/w emulsions enriched with 100 mg kg^−1^ of extract and of the control sample; (**b**) droplet size of the oil droplets measured in water and in SDS solution. Acronyms: leaves macerated (LM), leaves sonicated (LU), stems macerated (SM), stems sonicated (SU), bracts macerated (BM), and bracts sonicated (BU). Different letters indicate significant differences among samples (*p* < 0.05).

All extracts exhibited the ability to reduce surface tension compared to pure water at 20 °C, with a dose-dependent behavior. Since the extracts were characterized by different total phenolic contents [12], in a second step, the ST was compared at equal total phenolic content; a similar trend was observed, with BU showing the highest surface activity, while LU was the lowest. The SM and SU extracts, despite their high content of polyphenols, showed a very limited effect on the surface tension with the increasing of their concentration. Such extracts were characterized by high amounts of polar phenolic compounds like chlorogenic acid that, likely, partitioned mostly in the aqueous phase and thus had no main effects on the interfacial free energy. Similar findings were reported by Di Mattia et al. (2010) [21] for polar polyphenols like gallic acid. Notably, both LM and BM samples reached the lowest and comparable surface tension values at the highest concentrations tested; considering the significant difference in the TPC, it can be assumed that the phenolic pattern of the extracts is playing a major role, rather than the total phenolic content. Indeed, different glycosylated flavonoids were detected in the extracts in considerable amounts [12], luteolin-7-O-glucoside for the LM and apigenin-7-O-glucoside for BM, which, as observed by Caballero et al. (2022) [22], may exhibit interfacial properties due to the presence of a hydrophilic part (sugar) and a hydrophobic part (flavonoid backbone). After the surface properties, the extracts were used to investigate their behavior in o/w emulsions stabilized by a pea protein concentrate. The amount of proteins to be used for emulsion stabilization was obtained by investigating the ability of the pea protein concentrate to stabilize a 50% *w*/*w* sunflower oil-in-water emulsion. The minimum pea protein concentrate amount required to achieve a stable emulsion was found to be 4.6% *w*/*w* (expressed on the aqueous continuous phase). Regarding the extracts, a 100 mg kg^−1^ final concentration was selected for the enrichment; emulsions with no extract added were used as control samples. The effect of extract addition on the droplet size distribution is reported in Figure 2a while the droplet size of the oil particles is in Figure 2b. All the emulsions exhibited a bi-modal distribution (Figure 2a), showing a primary population of larger particles centered around 15 µm and a secondary population of smaller ones centered on 1 µm. The addition of LM and LU caused a shift in the particle size distribution that was reflected in larger droplet sizes (*p* < 0.05).

However, in the other cases, the addition of extract did not result in significant differences compared to the control neither in the droplet distribution nor in their size. The droplet size was measured also in SDS to investigate the presence of flocculation phenomena; from the results of particle size in SDS, it is evident that the presence of the extracts significantly increased the oil droplet dimension compared to the control (Figure 2b), although the particle size in SDS was significantly smaller than their counterpart in water, indicating the presence of flocculation in all the systems. Indeed, all the extracts significantly reduced the flocculation index from 105.44% ± 21.64% of the control to an average of 45.8% ± 16.01%, demonstrating the ability of the extracts to mitigate this phenomenon.

The use of pea proteins in emulsions can induce flocculation, a phenomenon widely documented in the literature [23,24]. This flocculation may contribute to the rapid destabilization of the system by accelerating gravitational separation, a generally undesirable outcome in emulsions [25]. Incorporating artichoke extracts thus showed promising results in modulating the flocculation index, effectively reducing flocculation without significantly altering particle size in most cases. Notably, an increase in particle size was observed only in emulsions containing leaf extracts compared to the control. The reduction in flocculation across all samples relative to the control may be attributed to the bioactive compounds in the extracts, primarily phenolics, which exhibit a high affinity for proteins, potentially leading to functional changes [26,27].

#### 2.1.2. Emulsions Flow Curves

**Figure 3:**
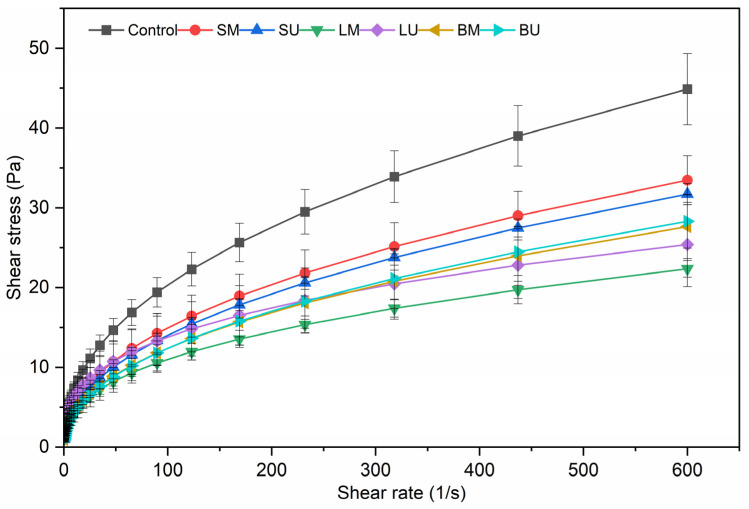
Flow curves of emulsions enriched with phenolic extracts compared to the control. Acronyms: leaves macerated (LM), leaves sonicated (LU), stems macerated (SM), stems sonicated (SU), bracts macerated (BM), and bracts sonicated (BU).

The flow curves of the emulsions are shown in Figure 3. All samples exhibit non-Newtonian flow behavior, likely due to the high concentration of sunflower oil (50% *w*/*w*), and this behavior does not appear to be affected by the presence of the extracts. The control emulsion demonstrated higher shear stress compared to the samples with added extracts. This behavior could be attributed to the increased flocculation in the control, which entrapped a more continuous phase [25]. The flow behavior curves were modeled with the Power Law and the fitted parameters of each emulsion are presented in Table 1. With the exception of sample LU, no significant (*p* ˂ 0.05) differences in the flow behavior index (n) between the enriched emulsions and the control were observed, while all samples exhibited pseudoplastic behavior. Regarding the consistency coefficient (k), all the samples showed no significant differences (*p* ˂ 0.05) with the control, except for the emulsions added with bracts extract (BM and BU) that were characterized by lower viscosity.

The effect of extracts rich in bioactive compounds on the viscosity of oil-in-water emulsions showed contrasting results in the literature. In a study by Di Mattia et al. (2014) [28], the addition of olive phenolic compounds to o/w emulsions stabilized by β-lactoglobulin did not significantly affect their flow behavior. In contrast, the incorporation of citrus extracts rich in flavonoids and limonoids was found to have a concentration-dependent influence on the viscosity of oil-in-water emulsions stabilized by a pea protein isolate, as a consequence of their effect on the dispersion state of the systems [17]. The discrepancies observed in the literature may be attributed to the complexity of the systems and to the varying effects of phenolic compounds on the colloidal state, including particle size and distribution, as well as their interactions with protein emulsifiers and other emulsion components. These interactions are likely to play a crucial role in determining the overall rheological properties of the emulsion systems.

#### 2.1.3. Oxidative Stability of Emulsions

**Figure 4:**
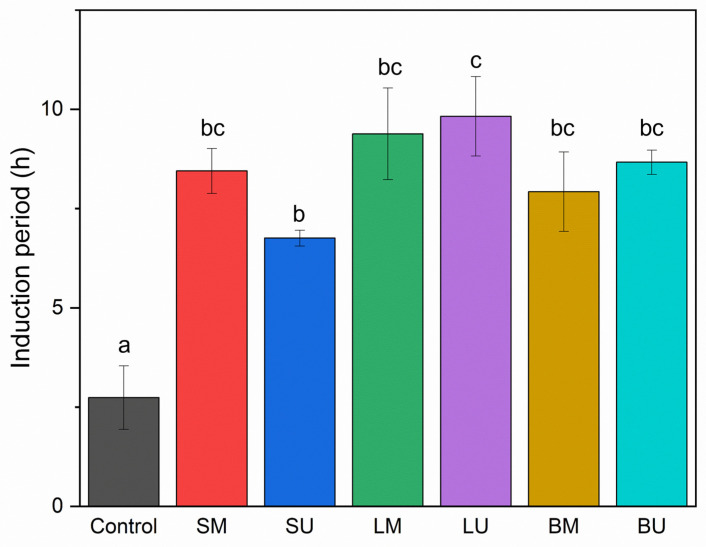
Induction period expressed in hours (h) of the control sample and of the emulsions enriched with phenolic-rich extracts. Acronyms: leaves macerated (LM), leaves sonicated (LU), stems macerated (SM), stems sonicated (SU), bracts macerated (BM), and bracts sonicated (BU). Different letters indicate significant differences among samples (*p* < 0.05).

As an additional chemical characterization, the emulsions underwent an accelerated oxidation test using the OxiTest method to assess the potential impact of the phenolic extracts on the matrix. This method quantifies the induction period (IP), which is the time required for the sample to start oxidation. A longer induction period indicates greater stability against oxidation over time [29]. The results of the oxidative test stability are reported in Figure 4. Under these conditions, the extracts demonstrated an improvement in the oxidative stability of the emulsions; indeed, the induction period (IP) value was significantly lower (*p* < 0.05) in the control compared to all other systems where the artichoke extracts were added. These results corroborate the findings of Cannas et al. (2023) [12] and demonstrate that such extracts can exert antioxidant activity even in complex multiphasic systems like oil-in-water emulsions, thereby showing a protective effect on oxidative phenomena. Indeed, all tested extracts exhibited significant antioxidant activity as all enriched systems showed higher IP compared to the control. The antioxidant capacity of artichoke extracts can be closely associated with the presence of flavonoids, which are recognized for their exceptional hydrogen-donating ability [30]; however, if the total content of phenolic and flavonoid compounds in the extracts is taken into consideration, it can be observed that there is no correlation with IP in the emulsions, indicating that the protective effect towards oxidation is likely to be more dependent on the specific phenolic pattern rather than the overall phenolic content. This may be explained by the fact that lipid oxidation in emulsified systems is a complex interfacial phenomenon, in which the antioxidant activity of a phenolic compound mainly depends on its nature and on its physicochemical properties in relation to its affinity for the oil/water interfaces [21]. The antioxidant capacity of artichoke extracts in complex multiphasic systems was also observed by Marques et al. (2017) [31], who investigated the ability of artichoke bioactive compounds to scavenge reactive oxygen species (ROS) both in o/w emulsions and in hydrogels formulated for cosmetic purposes.

### 2.2. Extract Encapsulation by Spray Drying, Exploitation in a Real Matrix, and In Vitro Digestion

Phenolic-rich extracts can be characterized by chemical and physical instability under conditions commonly encountered in processing and storage; at the same time, they can also deliver unpleasant sensory perceptions like bitterness, pungency, and off-flavors. Encapsulation technology can represent a valuable strategy to improve the overall stability of chemically sensitive extracts and mitigate any sensory off-taste/off-flavor as well as to modulate their gastrointestinal fate. Moreover, the encapsulation step was explored as a strategy to enhance the stability and controlled release of phenolic compounds in a real food matrix, linking the technological characterization of the extracts to their potential functional application. LM extract was selected as the system to be encapsulated by using spray-drying technology and maltodextrins as wall material; the choice was dictated by the chemical instability of such extract, which was evaluated in preliminary tests by the decrease in total phenolic content over time, as well as by practical considerations, leaves being the most abundant by-product generated by artichoke processing, along with stalks and roots (70–85%) [32]. Maltodextrins were chosen after preliminary tests, also taking into consideration their capacity to mask bitterness [33]. Indeed, artichoke leaves, according to Eljounaidi et al. (2015) [34], are rich in bitter compounds such as cynaropicrin, a compound typical of artichokes, that is proven to contribute approximately 80% to the bitter taste, and grosheimin that is present in smaller amounts [35].

Following the spray-drying process, an encapsulation efficiency of 90.21% ± 3.99% and a yield of 93.18% ± 3.99% were obtained; the moisture content of the powder was also assessed, revealing a narrow dry matter value of 96% ± 0.01, corresponding to a moisture content of about 4%.

**Figure 5:**
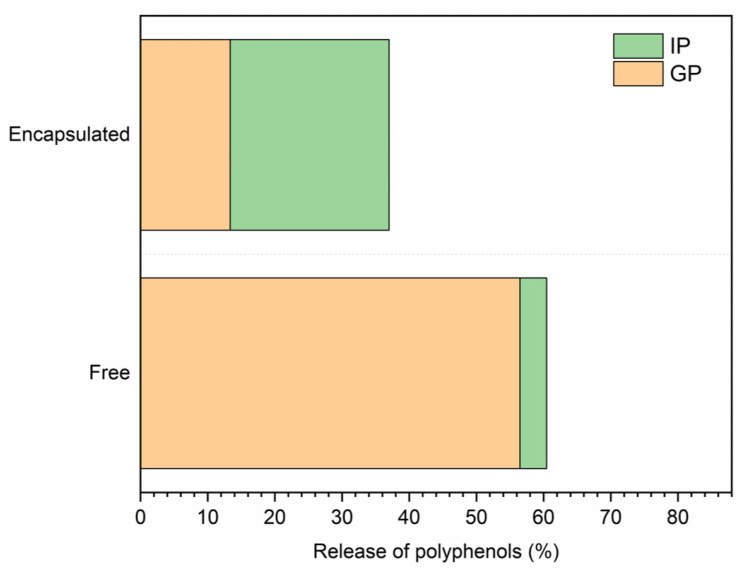
Recovery rates of polyphenols after in vitro digestion of a commercial vegan mayonnaise added with the artichoke leaves extract either as a free extract or as encapsulated. Acronyms: IP: Intestinal Phase; GP: Gastric Phase. Coefficient of variation: Free extract GP: 19.56%; Encapsulated extract GP: 19.12%; Free extract IP: 29.07%; Encapsulated IP: 25.35%.

Such encapsulated extract was used to enrich a commercial vegan mayonnaise and the effect of encapsulation was evaluated on the release of phenolic compounds under simulated digestion conditions; a sample of vegan mayonnaise enriched with the free extract, at equal concentration, was taken as a reference. Vegan mayonnaise with no extract added was taken as a blank system. Studies have suggested that encapsulated polyphenols may exhibit higher bioavailability compared to free polyphenols when evaluated in vitro [36]. The release of polyphenols was assessed by evaluating the total polyphenol content (TPC) after both the gastric and intestinal phases; the recovery rates were calculated starting from 375 mg kg^−1^, the concentration chosen for the post-process enrichment of the food matrix which corresponded to the initial concentration of polyphenols in the systems. The amount of encapsulated and free extract added was determined based on preliminary optimization tests which took into consideration system stability, sensory acceptability, and reliability of results. In Figure 5, the results concerning the recovery rates of polyphenols in both the gastric and intestinal phases are reported thus: the encapsulation process affected the release of phenolic compounds. In terms of total phenolic compounds released upon digestion, indeed, the percentage of polyphenols referred to the gastric phase was significantly higher (*p* ˂ 0.05) in the emulsions enriched with the free extract (56.48% ± 11.04%), compared to the mayonnaise in which the encapsulated extract was used (13.36% ± 2.55%). These results suggested that the release of polyphenols was limited when the sample was encapsulated because of the wall material, maltodextrins, which were resistant to the gastric environment characterized by very low pH equal to 2–3. Similar results were reported by Ćujić-Nikolić et al. (2019) [37], who studied the release of polyphenols from chokeberry after the encapsulation with maltodextrin and skimmed milk. The maltodextrin matrix was resistant to the gastric phase exhibiting a higher protective effect compared to skimmed milk. Comparable results were reported also by Stoica et al. (2022) [38] on anthocyanin extract from onion although in this last case, the extract was not encapsulated by using maltodextrins as wall material, but with a combination of Arabic gum and soy protein isolate.

Interestingly, the encapsulation affected the release also in terms of gastric-to-intestinal release ratio: in fact, the release of phenolic compounds upon intestinal digestion was significantly higher (19.49% ± 4.94%) in the encapsulated sample compared to the sample with the free extract (3.99% ± 1.16%). Thus, in the sample containing the free extract, which lacks a protective agent, the release of polyphenols predominantly occurred in the gastric environment. Conversely, in the sample enriched with the encapsulated extract, the encapsulation process provided protection to the phenolic compounds. These results corroborate the findings of González et al. (2020) [39], who demonstrated that encapsulating olive leaf extract with various materials, including maltodextrin, resulted in a higher oleuropein content in the encapsulated sample compared to the free extract after two hours of intestinal digestion. This implied the formation of a polysaccharide-polyphenol complex, which confers protective effects.

Although the total amount of released polyphenols was higher in samples with the free extract, such release predominantly occurred during the gastric phase. This premature release of polyphenols could pose challenges for their bio-accessibility, as they might undergo degrading transformations before reaching the intestinal phase, where they are considered to be absorbed. Indeed, a pre-requisite for polyphenols to exert any biological activity is that they must be bio-accessible in the gastrointestinal tract and subsequently absorbed in the small intestine to reach systemic circulation, as well as target tissues and organs within the body [40]. It is important to highlight that this study used the initial INFOGEST protocol to ensure comparability with previous research findings; however, the updated conditions introduced in INFOGEST 2.0 [41] could further enhance physiological relevance and should be considered in future studies.

## 3. Materials and Methods

### 3.1. Materials

The liquid extracts were derived from a previous study by Cannas et al. (2023) [12], which was conducted with the objective of maximizing the recovery of phenolic compounds from artichoke by-products (i.e., outer bracts, stems, and leaves). Specifically, the polyphenol-rich liquid extracts, obtained by using different ethanolic solutions and through two optimized extraction methods, maceration and ultrasound-assisted extraction (UAE), comprised the following: leaves macerated (LM), leaves sonicated (LU), stems macerated (SM), stems sonicated (SU), bracts macerated (BM), and bracts sonicated (BU). A comprehensive overview of the extraction conditions, previously described by Cannas et al. (2023) [12], is provided in Table 2. The sunflower oil was purchased in a local supermarket. The pea protein concentrate obtained by Dry Fractionation (DF) was kindly provided by S.A.L.P.A. (Roseto degli Abruzzi, Italy); according to the technical sheet, its composition was 53.1% proteins, 30.25% carbohydrates, 4.85% lipids, and 4.84 dietary fibers. Vegan mayonnaise was purchased in a local store (technical sheet: 55% lipids, 6.2% carbohydrates, 0.3% proteins, 1.6% salt). All the reagents were of analytical grade and were from Sigma-Aldrich and other equivalents.

### 3.2. Surface Tension of the Phenolic-Rich Extracts

The evaluation of air/water surface tension of artichoke phenolic extract solutions was carried out using a tensiometer (Sigma 700/701, Attention, Biolin Scientific, Espoo, Finland) equipped with a Wilhelmy platinum plate. The ethanolic extracts from Table 2 were tested at different increasing concentrations (0.1%, 0.25%, 0.5%, 1%, 2%, and 3% *v*/*v*) in the aqueous phase, on a final volume of 25 mL. The temperature of the measurements was set at 20 °C and the equilibration time was 30 min. Ethanol contribution to the surface tension of the diluted systems was checked and it was negligible.

### 3.3. Emulsion Preparation

Suspensions with 4.6% (*w*/*w*) of pea protein concentrate were prepared in ultrapure water and left to hydrate overnight. To prepare 50 g of oil-in-water emulsions (oil concentration 50% *w*/*w*), 25 g of sunflower oil was gradually mixed with 25 g of the previously described pea protein aqueous suspension added with the phenolics-rich extracts (final concentration of 100 ppm on the aqueous phase). The pre-homogenization step was performed with a rotor-stator device (YellowLine DI 25 Basic, IKA Werke GmbH & Co, Staufen im Breisgau, Germany) set at a speed of 13,500 rpm for 120 s. The homogenization step was carried out with a high-pressure homogenizer (Panda Plus 2000, GEA Niro Soavi, Parma, Italy) set at 300 bars for five cycles. Seven kinds of emulsions were prepared, one for each extract plus the control samples with no extract added (control). For each system, three independent replicates were prepared. Ethanol contribution to the emulsifying capacity was checked and it was negligible.

### 3.4. Emulsion Particle Size Distribution

The size and distribution of oil droplets in the o/w model emulsions were analyzed by static light scattering (Mastersizer 3000, Malvern Instruments Ltd., Worcestershire, UK). Emulsions were added to the dispersion unit (Hydro 2000S, Malvern Instruments Ltd., Worcestershire, UK) in distilled water at 2000 rpm to reach an obscuration between 5 and 6%, as evaluated with preliminary tests. For sunflower oil, a particle refractive index of 1.474 with particle adsorption of 0.01 was chosen, while for water a refractive index of 1.330 was used. Droplet size measurements are reported as the volume-weighted mean diameter D[4,3] and particle size distribution. A flocculated state of oil droplets was obtained, diluting the emulsions in a 1% (*w*/*v*) SDS solution, as described by D’Alessio et al. (2022) [42]. The flocculation index % (FI%) was then calculated as follows (Equation (1)):

(1) $$FI\%=\left(D{\left[4,3\right]}_{h2o}/D{\left[4,3\right]}_{SDS}-1\right)\times 100$$

where *D*[4,3]*_h_*_2*o*_ is the volume-weighted mean diameter measured in deionized water, while *D*[4,3]*_SDS_* is the volume-weighted mean diameter of the same sample diluted in 1% (*w*/*v*) *SDS* solution and then measured in deionized water.

### 3.5. Emulsions Flow Behavior

The emulsions were analyzed by a controlled stress-strain rheometer (MCR 302, Anton Paar, Graz, Austria). The instrument worked with a controlled bath temperature set at 20 °C and it used a flat-plate geometry. Flow curves were measured by recording shear stress (Pa) at a logarithmic increasing shear rate from 0.3 to 600 s^−1^. Data were fitted to the Ostwald de Waele (known as Power Law) (Equation (2)):

(2) $$\tau =K{\left(\dot{\gamma }\right)}^{n}$$

where: *τ* is the shear stress (Pa); *K* is the consistency coefficient (Pa·s^n^), a measure of apparent viscosity; $\dot{\gamma }$ is the shear rate (s^−1^); n is the flow behavior index (dimensionless).

### 3.6. Oxidative Stability Evaluation

The oxidative stability of the emulsions enriched with phenolic extracts and of the control emulsion was tested using the OXITEST Device (Velp Scientifica, Usmate, MB, Italy). Ten grams of o/w emulsion was weighed into the sample cells with a uniform distribution. The device temperature was set at 90 °C and the oxygen pressure was set at 6 bar [29]. The oxidative stability values of the samples were determined by the Oxisoft^TM^ version 5.0.2 software and reported as induction period (IP) values, corresponding to the absolute oxygen pressure drop inside the instrument chambers, due to the oxidation reaction, automatically calculated from the oxidation curve by the graphical method, based on two tangent methods.

### 3.7. Extract Encapsulation by Spray-Drying

Before encapsulation, the extract was submitted to rotary evaporation (Buchi R-100) at 40 °C and 30 mbar until the extraction solvent was completely removed and then solubilized with the same amount of deionized water. The extract was then freeze-dried using a Labogene (Allerød, Denmark) Scanvac Coolsafe freeze-dryer. The freeze-drying process was carried out at 0.316 hPa at −40 °C for 24 h and allowed to obtain a powdered extract. An aqueous solution was prepared by dissolving the artichoke powder extract and coating materials, either maltodextrin (MD) with Dextrose Equivalent (DE) of 7.5–9.9, or gum Arabic (GA), in a weight ratio of 1:5 (*w*/*w*). They were left under continuous stirring for 30 min at room temperature. The encapsulation by spray-drying was performed using a mini spray-dryer (Büchi Mini Spray-dryer B 290, Labortechnik AG, Flawil, Switzerland). The inlet air temperature was set at 150 °C, outlet was 85 °C with a feed rate of 9 mL min^−1^. At the end of the process, the powders were weighted, and the final yield was calculated.

### 3.8. Encapsulation Efficiency and Load Yield of Total Phenolic Content

The chemical characterization of the encapsulated powdered extract included the evaluation of the total polyphenol content (TPC) and the encapsulation efficiency that was assessed by evaluating the surface phenolic content (SPC) of the encapsulated extract and the load yield of total phenolic content, as detailed below.

For the evaluation of TPC, the procedure reported by Tatasciore et al. (2023) [43] was followed. A preliminary step of solubilization of the powders was necessary. Briefly, 0.2 g of each encapsulated extract was weighed and dissolved in 3 mL of distilled water. The powders were subjected to 5 min of ultrasound treatment using an ultrasonic bath (LAB SONIC LBS1 3Lt 2015, Falc Instruments, Treviglio, Bergamo, Italy) to achieve complete solubilization, followed by 30 s of vortexing. An extraction step was then performed to remove the carrier material from the sample. Specifically, 250 μL of the suspension was added to 250 μL of distilled water and 1 mL of extraction solvent (50:42:8, ethanol:water:acetic Acid), vortexed for 30 s, and centrifuged (Neya 16R refrigerated high speed) for 10 min at 6000 rpm at 10 °C. The supernatant was recovered and appropriately diluted for the TPC assay as reported in the extract chemical stability section. For the determination of the surface phenolic content (SPC), the procedure described by Ravichai and Muangrat (2019) [44] was followed: 10 mg of encapsulated artichoke powder was weighed and resuspended in 1 mL of ethanol:methanol (1:1). The mixture was vortexed for 1 min and centrifuged at 6000 rpm for 10 min (Neya 16R refrigerated high speed). The phenolic content of the SPC fraction was analyzed using the Folin-Ciocolteau method as reported in the extract chemical stability section.

The formula applied to calculate the Encapsulation Efficiency (EE%) was Equation (3):

(3) $$\;\;EE\;\left(\%\right)=(Total\;phenolic\;content\;\left(mgGAE/g\;powder\right)-Surface\;phenolic\;\;\;content\;\left(mgGAE/g\;powder\right)/Total\;phenolic\;content\;\left(mgGAE/g\;powder\right)\times 100$$

The load yield (Y) which indicated the total phenolic content (TPC) present in the encapsulated powders after the process was calculated using the following formula (Equation (4)):

(4) $$Y\;\left(\%\right)=\left(Total\;phenolic\;content\;\left(mgGAE/g\;powder\right)\right)/\left(Calculated\;value\;of\;added\;phenolic\;\left(mgGAE/g\;dry\;extract\right)\right)\times 100$$

### 3.9. Determination of Dry Matter of the Encapsulated Powdered Extract

The moisture content of the artichoke’s encapsulated powder was determined gravimetrically by drying the samples in an oven at 105 °C until constant weight.

### 3.10. Enrichment of Commercial Vegan Mayonnaise

For the enrichment of the commercial vegan mayonnaise, the extract was added in the encapsulated form (obtained through spray-drying) or in the free form; in this last case, the liquid extract was preliminarily submitted to freeze-drying and was thus in a solid form. The TPC in the final samples was set to 375 mg kg^−1^.

### 3.11. In Vitro Digestion

Digested emulsion-enriched encapsulated artichoke extract samples were obtained according to the harmonized INFOGEST static in vitro digestion procedure, simulating the physiological conditions of the oral, gastric, and small intestinal digestion phases in vitro [45] with some modifications. The oral phase was performed using human saliva instead of simulated oral fluid. Saliva was collected from healthy volunteers, according to Chen et al. (2018) [46]. The fresh saliva samples were collected after 2 h from the last meal. The donors were invited to rinse their mouths with deionized water for at least 30 s to obtain a neutral environment, and then saliva at the first 30 s was discarded. Saliva was collected in the next 5 min each 30 s, until the needed amount was reached. The collected saliva was immediately centrifuged at 5000 rpm for 10 min and the supernatant was stored at −20 °C. To simulate mastication, 1 mL of human saliva was added to 1 g of emulsion enriched with encapsulated artichoke extract, and then the mixture was ground with mortar and pestle for 2 min; then, the final volume was made up to 2 mL with deionized water. The gastric phase was started by adding simulated gastric fluid containing pepsin (2000 U/mL of activity in the final volume). The pH was adjusted to 3 and the volume to 4 mL, and the mixture was incubated at 37 °C for 2 h in a rotating mixer. Then, a solution containing simulated intestinal fluid, containing bile extract (10 mM of bile salts in the final volume) and pancreatin (100 U/mL of trypsin activity in the final volume) was added. The pH was adjusted to 7 and the volume to 8 mL, and the mixture was incubated 2 h at 37 °C in a rotating mixer. At the end of digestion, digesta was collected for TPC evaluation. Before the analysis, the digested samples were treated with Trichloroacetic acid (TCA), as reported by Bu et al. (2022) [47] to precipitate proteins to avoid interferences during TPC measurements. Thus, TCA was added in all the samples (to get a 5% (*v*/*v*) concentration in the system) after 2 h of both the gastric and the intestinal phase. These solutions were centrifuged at 4000 rpm for 20 min; the supernatant was taken, neutralized, filtered through 0.45 µm filters, and used for TPC determination

### 3.12. Statistical Analysis

All the experiments were carried out in triplicate (n = 3). Results were reported as mean and standard deviation. A one-way analysis of variance (ANOVA) and Tukey’s test were used to establish the significance of differences at the 0.05 significance level. Data analysis was carried out by using Origin (Pro) Version number 2022 (OriginLab Corporation, Northampton, MA, USA).

## 4. Conclusions

In this study, the technological properties of six ethanolic phenolic-rich extracts obtained from artichoke bracts, stems, and leaves for the formulation of oil-in-water emulsions were explored along with encapsulation by spray-drying for exploitation in a real emulsified matrix. The effect of encapsulation on extracts polyphenols bioavailability in a commercial vegan mayonnaise was also investigated. All the extracts were proven to exert surface properties in aqueous solutions. When tested in emulsions, the addition of artichoke extracts did not cause any significant effect on the droplet size, with the exception of leaves extracts, which caused a shift of the droplet populations towards larger size; however, in all the systems, the artichoke extracts were able to mitigate flocculation phenomena, which are quite common when pea proteins are used as emulsifiers. No significant effects were found on sample flow behavior, while a significant improvement in oxidative stability occurred as a consequence of extract enrichment. The encapsulation by spray-drying of the leaf extract by using maltodextrins as wall material affected the release of phenolic compounds from a commercial vegan mayonnaise submitted to simulated gastro-intestinal digestion, showing a protecting effect towards the gastric environment and a prevalent release during the intestinal phase, thus potentially favoring their absorption and bio-accessibility. Considering the necessity to improve the sensory properties of the extracts and reduce the bitterness perception, further studies are necessary; to this aim, it would be interesting to explore the utilization of either other encapsulating agents or encapsulation techniques.

## Figures and Tables

**Table 1:** Power law parameters k (consistency coefficient) and n (flow index) of the o/w emulsions enriched with artichoke by-product extracts (100 mg kg^−1^) and of the control sample with no added extract taken as reference. Acronyms: leaves macerated (LM), leaves sonicated (LU), stems macerated (SM), stems sonicated (SU), bracts macerated (BM), and bracts sonicated (BU). R^2^ is the coefficient of determination indicating the goodness of fit of the model.

Sample	k	n	R^2^
Control	2.692 ± 0.432 ^a^	0.441 ± 0.028 ^a^	0.999 ± 2.89 × 10^−4^
SM	1.943 ± 0.716 ^ab^	0.454 ± 0.052 ^a^	0.999 ± 8.29 × 10^−5^
SU	1.732 ± 0.121 ^ab^	0.455 ± 0.01 ^a^	0.999 ± 1.36 × 10^−4^
LM	1.808 ± 0.392 ^ab^	0.396 ± 0.032 ^ab^	0.999 ± 2.63 × 10^−4^
LU	2.899 ± 0.853 ^a^	0.342 ± 0.017 ^b^	0.999 ± 2.68 × 10^−4^
BM	1.601 ± 0.586 ^b^	0.451 ± 0.032 ^a^	0.999 ± 4.39 × 10^−5^
BU	1.489 ± 0.255 ^b^	0.461 ± 0.006 ^a^	0.999 ± 5.25 × 10^−5^

Values are means ± standard deviation. Within the same column, different letters indicate significant differences among samples (*p* < 0.05).

**Table 2:** Optimized extraction conditions were employed to obtain artichoke by-products of liquid ethanolic extracts by Maceration (M) and Ultrasound-Assisted Extraction (UAE), along with Total Phenolic Content (TPC, mg/g_dm_, mg per g of dried matter). More detailed information is reported in the work by Cannas and co-authors [12]. Acronyms: leaves macerated (LM), leaves sonicated (LU), stems macerated (SM), stems sonicated (SU), bracts macerated (BM), and bracts sonicated (BU).

Method	By-Product	Legend	Parameters				TPC (mg/g_dm_)
			Frequency (Hz)	Power (W)	Ethanol (%)	Time (min)	
M	Stems	SM			53	60	2604 ± 10 ^a^
	Leaves	LM			45	60	1863 ± 6 ^d^
	Bracts	BM			50	60	1866 ± 5 ^d^
UAE	Stems	SU	40	144	42	10	2516 ± 4 ^b^
	Leaves	LU			20	10	1723 ± 20 ^e^
	Bracts	BU			64	41	2014 ± 31 ^c^

Different letters indicate significant differences among samples (*p* < 0.05).

## Data Availability

The original contributions presented in the study are included in the article. Further inquiries can be directed to the corresponding authors.
